# Multi-Level Physiological and Metabolomic Responses of *Hypnum plumaeforme* Reveal Oxidative Imbalance, Photosynthetic Disruption and Metabolic Reprogramming Under Cesium Stress

**DOI:** 10.3390/plants15162504

**Published:** 2026-08-19

**Authors:** Siyu Sun, Binjie Zhou, Xin Liu, Mingyi Qin, Xinyan Sun, Min Wang, Peng Ren, Ke Chen

**Affiliations:** 1 College of Life Science and Agri-Forestry, Southwest University of Science and Technology, Mianyang 621010, China; ssy@mails.swust.edu.cn (S.S.); zhoubj@mails.swust.edu.cn (B.Z.); 2255114_la@mails.swust.edu.cn (X.L.); qmy20241618@mails.swust.edu.cn (M.Q.); sunxy@mails.swust.edu.cn (X.S.); wangmin@mails.swust.edu.cn (M.W.); 2 Tianfu Institute of Research and Innovation, Southwest University of Science and Technology, Chengdu 610213, China

**Keywords:** cesium stress, moss, antioxidant physiology, chlorophyll fluorescence, response mechanisms

## Abstract

Cesium (Cs) contamination has become an increasing environmental concern because of its high mobility, persistence, and ecological risks. Mosses have attracted considerable attention as bioindicators owing to their high environmental sensitivity; however, their responses to Cs remain poorly understood. In this study, the moss *Hypnum plumaeforme* was exposed to Cs^+^ (5, 50 and 500 mg L^−1^) stress, and its morphological characteristics, antioxidant responses, photosynthetic performance and metabolomic alterations were comprehensively investigated. Low Cs^+^ exposure induced early oxidative signaling by increased H_2_O_2_ accumulation and slight stimulation of photosynthesis, whereas moderate stress triggered pronounced superoxide production and activation of antioxidant defenses. High Cs^+^ exposure caused severe cellular deformation, disruption of photosystem II, excessive hydroxyl radical accumulation, and collapse of the coordinated antioxidant–osmotic regulatory network. Untargeted LC–MS metabolomics further revealed substantial metabolic reprogramming under severe stress, including inhibition of carbon metabolism, significant enrichment of tryptophan metabolism, and accumulation of defensive secondary metabolites, indicating a shift in metabolic resources from growth toward stress defense. Collectively, these findings demonstrate the physiological responses of *H. plumaeforme* to Cs^+^ in a concentration-dependent manner. The high sensitivity of oxidative biomarkers, chlorophyll fluorescence parameters and metabolic signatures highlights the potential of *H. plumaeforme* as a bioindicator for assessment of cesium contamination.

## 1. Introduction

Cesium contamination poses relatively severe environmental concerns. Cesium is an extremely reactive alkali metal, exhibiting chemical properties similar to those of sodium (Na) and potassium (K), and possesses high water solubility [1]. The sources of cesium contamination are complex and varied, arising from the natural weathering release of cesium-bearing minerals such as pollucite, celestite, and strontianite, and from anthropogenic activities including nuclear power plant accidents, discharge of nuclear wastewater, loss of radioactive sources in medical and industrial applications, combustion of cesium-containing coal, and improper disposal of solid wastes. Through means such as surface water irrigation, atmospheric dry and wet deposition, and solid waste stockpiling, these sources lead to the persistent accumulation of cesium in the environment, posing severe threats to ecosystem stability and human health [1,2,3]. Although radioactive isotopes are of particular concern, the physiological effects of stable Cs^+^ (^133^Cs) on plants also warrant investigation. Thus, developing cost-effective and environmentally friendly monitoring technologies for cesium contamination is of practical significance.

Although traditional physical and chemical monitoring methods are effective to some degree, they generally suffer from limitations such as high cost, complex operation, and the risk of secondary pollution, limiting large-scale, long-term, and dynamic monitoring. Plant-based monitoring techniques have attracted widespread attention owing to their simplicity and cost-effectiveness; among these, mosses are extensively used to assess contamination levels of heavy metals and radionuclides, thanks to their unique biological characteristic [4,5]. Mosses lack true roots and vascular tissues; they possess small cells (approximately 10–30 μm in diameter) with thin cell walls, and their entire surface can efficiently accumulate elements such as U and Sr through ion exchange and physical adsorption, and even retain a certain adsorption capacity after tissue death [6,7,8]. Simultaneously, mosses grow slowly, are widely distributed and structurally simple, and lack a cuticle and stomata, which allows atmospheric deposition and soil pollutants to penetrate directly through the cell walls and enter the plant body, resulting in long-term continuous accumulation [4,9]. In terms of ecological risk assessment, mosses exhibit high physiological sensitivity to stress from heavy metals and radionuclides [10], and this sensitivity can directly reflect the degree of stress imposed by pollutants on biological systems. Moreover, mosses show strong adaptability to oligotrophic environments and are capable of colonizing various substrates, including rocks, bark, and soils [7]. These characteristics make them ideal bioindicators that combine representativeness, sensitivity, and practical applicability.

Among the diverse moss groups, the genus *Hypnum* is widely distributed and has been employed for environmental pollution monitoring worldwide. Among them, *Hypnum plumaeforme*, which is widely distributed in temperate and subtropical regions, possesses characteristics such as simple structure, sensitive responsiveness, strong cation exchange capacity, low cost, and environmental friendliness. These traits confer efficient accumulation and immobilization capacity, adaptability to nutrient-poor and stressful environments, and stress tolerance, indicating considerable application potential in environmental monitoring [11]. Recent studies have confirmed that *H. plumaeforme* exhibits strong absorptive accumulation capacity for atmospherically deposited metals such as Cd, Pb, Cu, Zn, and Cr; moreover, the species’ exposure to Pb and Ni induces dose-dependent ROS accumulation with synergistic oxidative effects [12,13]. Regarding radionuclides, post-Fukushima surveys have demonstrated high levels of ^134^Cs and ^137^Cs in *H. plumaeforme*, and their contents were correlated with ambient radiation and soil contamination, supporting its use as a qualitative indicator of radiocesium pollution [11]. Additionally, *H. plumaeforme* has been found to enrich ^210^Po and ^210^Pb in soils near uranium tailings [14]. However, although *H. plumaeforme* has demonstrated stable accumulation capacity for various heavy metals and radionuclides, systematic investigations into its multi-level physiological and metabolomic responses under cesium stress are still lacking, limiting its application in cesium contamination monitoring and risk assessment. Previous studies have primarily focused on *H. plumaeforme* radiocesium content with environmental contamination levels without addressing the underlying physiological and metabolic mechanisms. To address this, the present study exposed *H. plumaeforme* to three concentrations of Cs^+^ stress (5, 50, and 500 mg·L^−1^) and systematically analyzed its morphological phenotypes, antioxidant physiology, photosynthetic characteristics, and metabolomic profiles to elucidate the physiological and biochemical response patterns under Cs^+^ stress and to verify its suitability as an indicator plant for cesium contamination.

## 2. Results

### 2.1. Effects of Cs^+^ Stress on Morphological and Cellular Characteristics of H. plumaeforme

**Figure 1:**
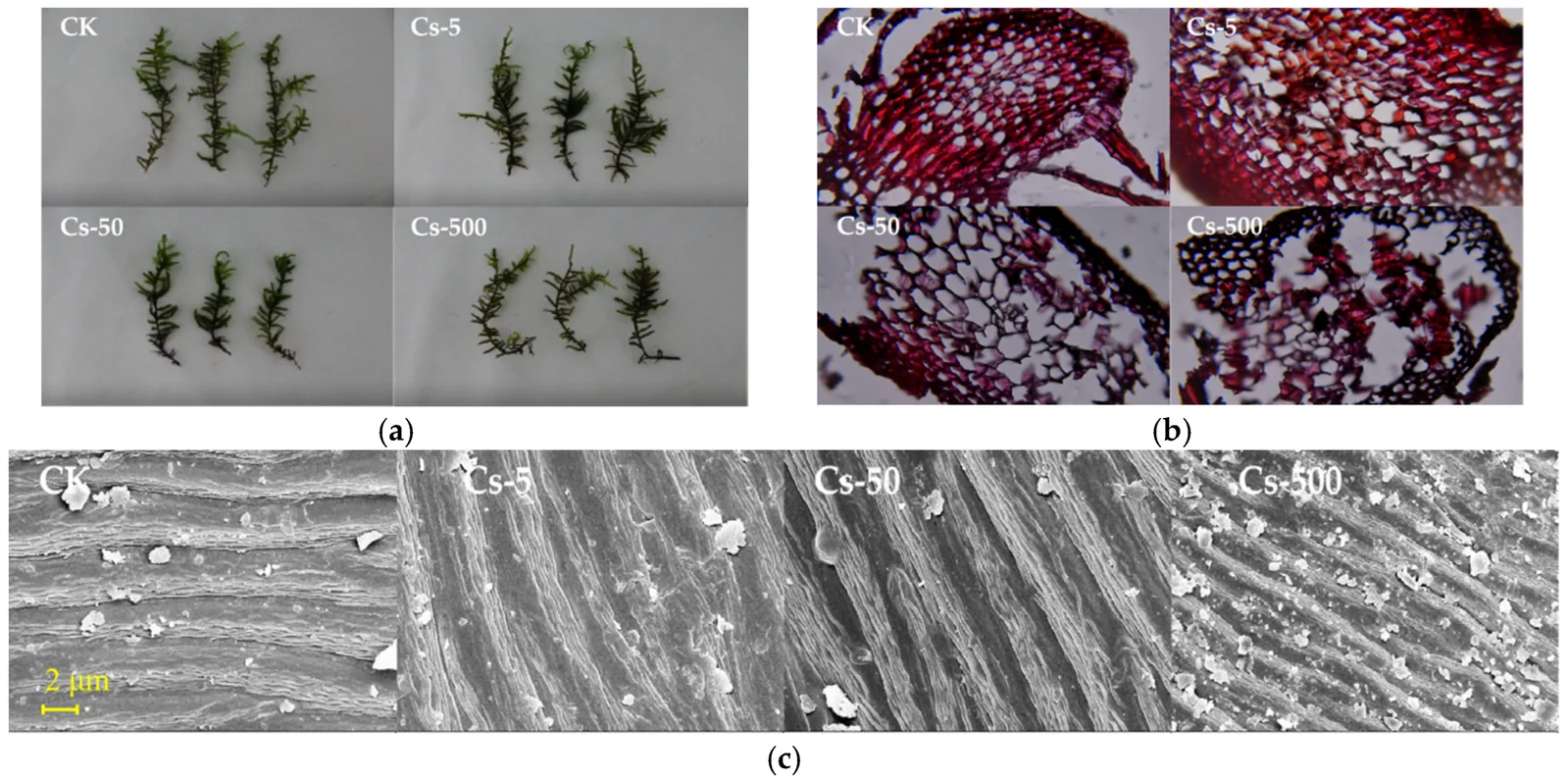
Morphological and cellular characteristics of *H. plumaeforme* under varying concentrations of cesium stress: (**a**) growth status; (**b**) paraffin sections of stem tissues; (**c**) scanning electron microscopy (SEM) micrograph (scale bar = 2 μm).

Morphological damage to *Hypnum plumaeforme* exhibited clear concentration-dependent differences among the treatment groups. In the 5 mg·L^−1^ Cs^+^ treatment group, the plants remained dense with dark green leaves (Figure 1a); stem cross-sections showed regularly arranged cells with clearly defined cell walls (Figure 1b). Overall morphology and cellular structure exhibited no obvious changes compared with the CK group, and scanning electron microscopy (SEM) revealed intact leaf textures (Figure 1c), with only occasional slight wrinkling observed in a few fields of view. In the 50 mg·L^−1^ treatment group, the stems and branches of *H. plumaeforme* exhibited pronounced atrophy. Paraffin sections indicated that stem cells became loosely arranged, with notable cell wall disruption, denser surface textures, and deeper folds. In the 500 mg·L^−1^ treatment group, the atrophy of stems and branches was further exacerbated, accompanied by extensive cellular disintegration and cavity formation. SEM observations revealed that the regular striated textures on leaf surfaces became denser and severely wrinkled, while the groove structures on stems and branches were notably deepened, collectively indicating a substantial aggravation of morphological injury.

### 2.2. Effects of Cs^+^ Stress on Antioxidant Responses of H. plumaeforme

#### 2.2.1. Changes in ROS, Membrane Lipid Peroxidation, and Antioxidant Enzyme Activities

**Figure 2:**
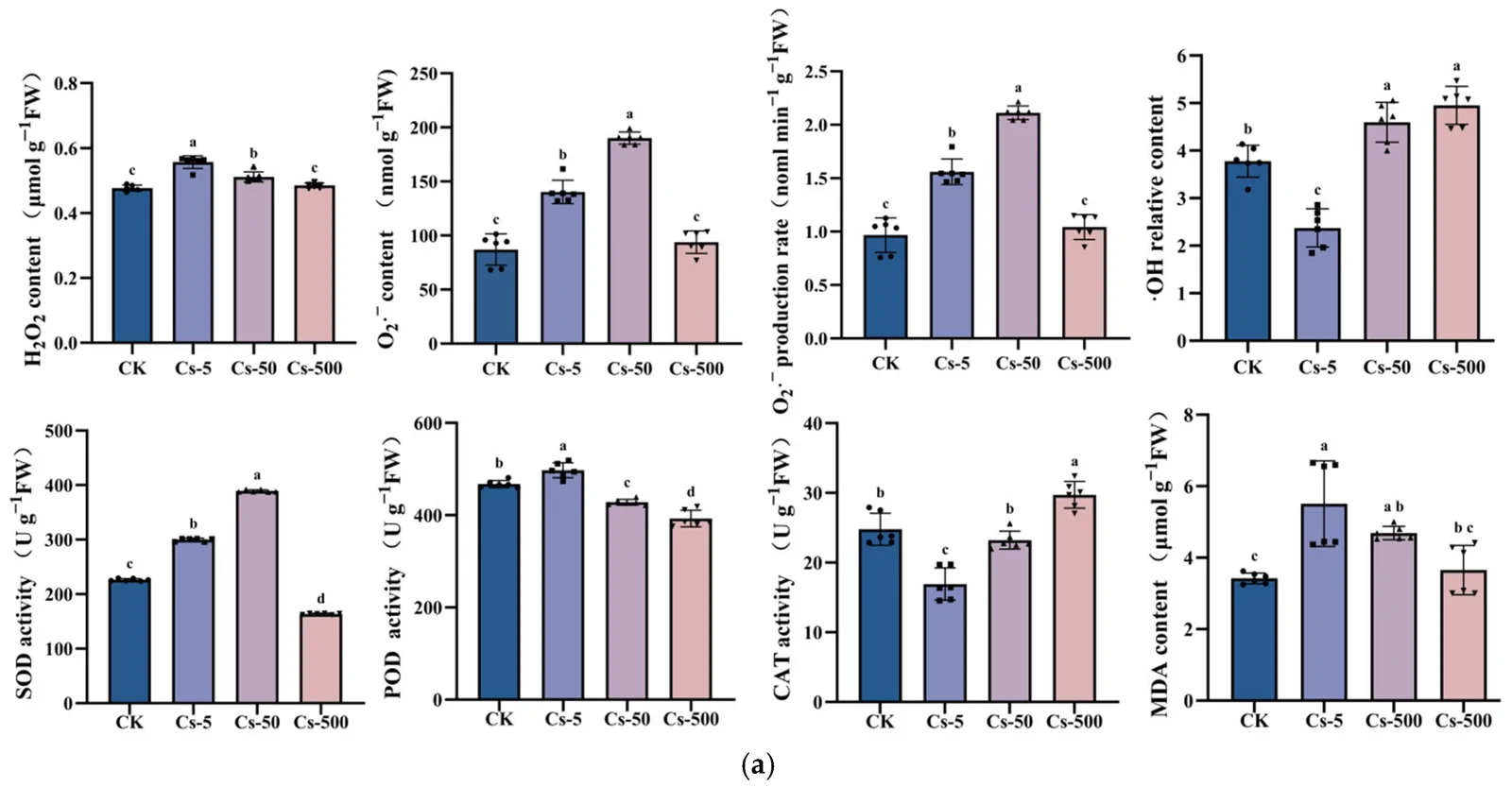

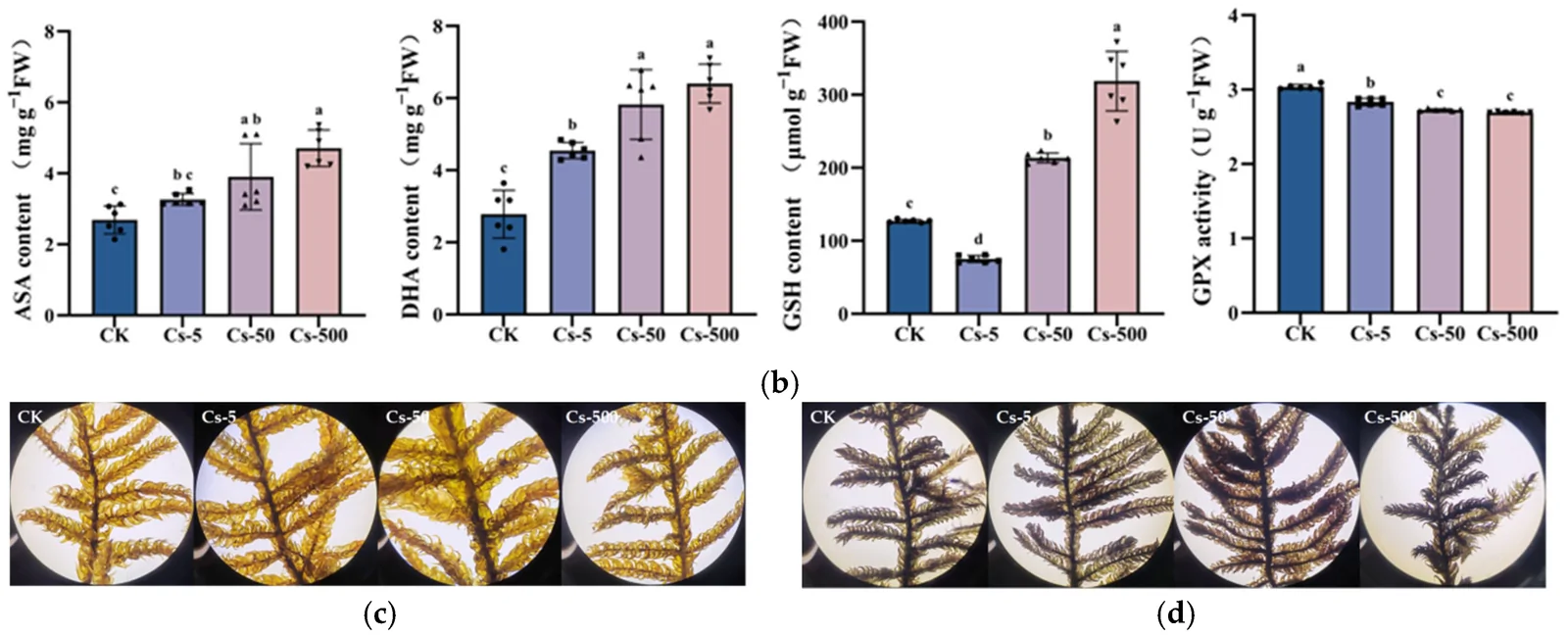
Antioxidant physiological responses of *H. plumaeforme* under varying concentrations of cesium stress: (**a**) Changes in ROS, membrane lipid peroxidation, and antioxidant enzyme activities. (**b**) Changes in enzymes and metabolites of the ASA-GSH cycle. (**c**) H_2_O_2_ staining (DAB). (**d**) O_2_·^−^ staining (NBT). Data are mean ± SD (*n* = 6). Different lowercase letters above the bars indicate significant differences among treatments (*p* < 0.05, one-way ANOVA with LSD post hoc multiple comparison).

Under Cs^+^ stress, the reactive oxygen species (ROS) content and antioxidant enzyme activities in *H. plumaeforme* exhibited concentration-dependent differences among the CK, Cs-5, Cs-50, and Cs-500 groups, with each parameter showing distinct response patterns across the treatment levels (Figure 2a). Regarding ROS, the hydrogen peroxide (H_2_O_2_) content was highest in the Cs-5 group among all treatments at 0.557 μmol·g^−1^FW, representing a significant increase of 16.8% compared with the control (CK) group (*p* < 0.05). In contrast, the superoxide anion (O_2_·^−^) content and its production rate were highest in the Cs-50 group compared with CK and other treatments, with an increase of 118% relative to CK, while both parameters returned to control levels in the Cs-500 group. Meanwhile, the 3,3′-Diaminobenzidine (DAB) and the Nitroblue Tetrazolium (NBT) tissue staining results were consistent with the quantitative trends described above, visually demonstrating the deepest H_2_O_2_ staining in the Cs-5 group and the deepest O_2_·^−^ staining in the Cs-50 group (Figure 2c,d). The relative content of hydroxyl radicals (·OH) was lowest in the Cs-5 group, showing a significant reduction of 37.1% compared with CK, but significantly higher in the Cs-50 and Cs-500 groups than in CK by 21.8% and 31.2%, respectively. In terms of membrane lipid peroxidation, the malondialdehyde (MDA) content followed a trend generally consistent with the accumulation of H_2_O_2_ and O_2_·^−^, peaking in the Cs-5 group with a significant increase of 61.2% over CK, after which it declined progressively with increasing Cs^+^ concentrations. Regarding antioxidant enzyme activities, superoxide dismutase (SOD) activity peaked in the Cs-50 group at 389.340 U·g^−1^FW, representing a significant increase of 72.0% over CK, then decreased to 163.685 U·g^−1^FW in the Cs-500 group, a reduction of 27.7% relative to CK. This trend coincided with the concurrent maximum response of O_2_·^−^ observed in the Cs-50 group. Peroxidase (POD) activity reached its maximum in the Cs-5 group, with a 6.4% increase over CK, and then declined continuously with increasing Cs^+^ concentration, falling to 392.766 U·g^−1^FW in the Cs-500 group, a decrease of 16.0% relative to CK. In contrast, catalase (CAT) activity exhibited an opposite trend, reaching its lowest level in the Cs-5 group with a 31.7% decrease from CK, but was induced and activated under high-concentration stress, showing a 19.9% increase over CK.

#### 2.2.2. Changes in Enzymes and Metabolites of the ASA-GSH Cycle

Under varying concentrations of Cs^+^ stress, the contents of non-enzymatic antioxidants and the activities of related enzymes in the ascorbate-glutathione (ASA-GSH) cycle of *H. plumaeforme* exhibited differential changes (Figure 2b). Ascorbic acid (ASA) content increased continuously with rising Cs^+^ concentration, with values of 2.691, 3.266, 3.904, and 4.708 mg·g^−1^FW in the CK, Cs-5, Cs-50, and Cs-500 groups, respectively. The increases in the Cs-50 and Cs-500 groups were 45.1% and 74.9% relative to CK, respectively, both reaching statistical significance (*p* < 0.05). Dehydroascorbic acid (DHA) content followed a trend similar to that of ASA, with values of 2.781, 4.546, 5.823, and 6.404 mg·g^−1^FW across the four groups. The Cs-50 and Cs-500 groups showed significant increases of 109% and 130% over CK, respectively. Reduced glutathione (GSH) content exhibited a decreasing-then-increasing trend, reaching its lowest value (75.138 μmol·g^−1^FW) in the Cs-5 group, representing a significant decrease of 41.0% compared with CK. Subsequently, it rebounded to 213.552 and 318.759 μmol·g^−1^FW in the Cs-50 and Cs-500 groups, respectively, with the Cs-500 group showing a significant increase of 150% relative to CK. Glutathione peroxidase (GPX) activity was significantly lower in the Cs-50 and Cs-500 groups than in the CK group, with values of 3.034, 2.834, 2.728, and 2.694 U·g^−1^FW in the four groups, corresponding to an 11.2% reduction in the Cs-500 group relative to CK. Under high-concentration Cs^+^ stress, the contents of ASA, DHA, and GSH in *H. plumaeforme* all reached their maximum levels, whereas GPX activity remained continuously suppressed.

#### 2.2.3. Changes in Osmotic Adjustment Substances

The contents of osmotic adjustment substances in *H. plumaeforme* also exhibited concentration-dependent variations among the CK, Cs-5, Cs-50, and Cs-500 groups (Table 1; *p* < 0.05). Soluble protein content showed a decreasing-then-increasing trend. Compared with CK, it decreased by 6.6% in the Cs-5 group, then rebounded to 6.517 mg·g^−1^FW in the Cs-50 group, and further increased to 6.865 mg·g^−1^FW in the Cs-500 group, representing a 9.2% increase over CK. Soluble sugar content exhibited an increasing-then-decreasing trend. It rose to 72.606 mg·g^−1^FW in the Cs-5 group, a 12.7% increase over CK, peaked at 103.652 mg·g^−1^FW in the Cs-50 group with a significant increase of 60.9% relative to CK, and then declined to 76.129 mg·g^−1^FW in the Cs-500 group, though it remained above the CK level. Free proline content accumulated continuously with increasing Cs^+^ concentration, with values of 6.651, 7.409, 14.563, and 18.848 μg·g^−1^FW across the four treatment groups. The Cs-50 and Cs-500 groups showed significant increases of 119.0% and 183.4% over CK, respectively. Under Cs^+^ stress, soluble protein and free proline in *H. plumaeforme* accumulated continuously as osmotic adjustment substances, whereas soluble sugar first increased and then decreased.

### 2.3. Effects of Cs^+^ Stress on Photosynthetic Responses of H. plumaeforme

**Figure 3:**
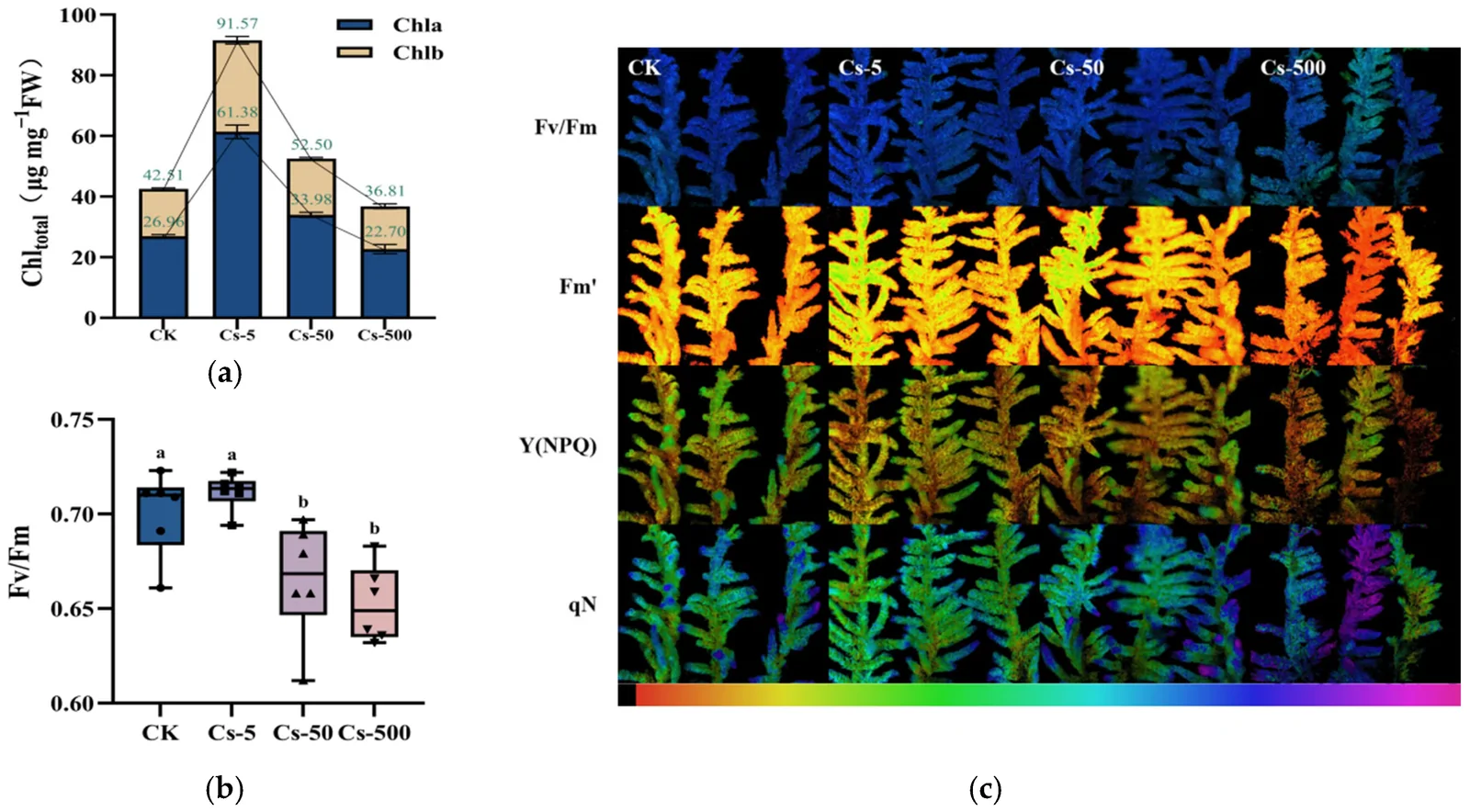

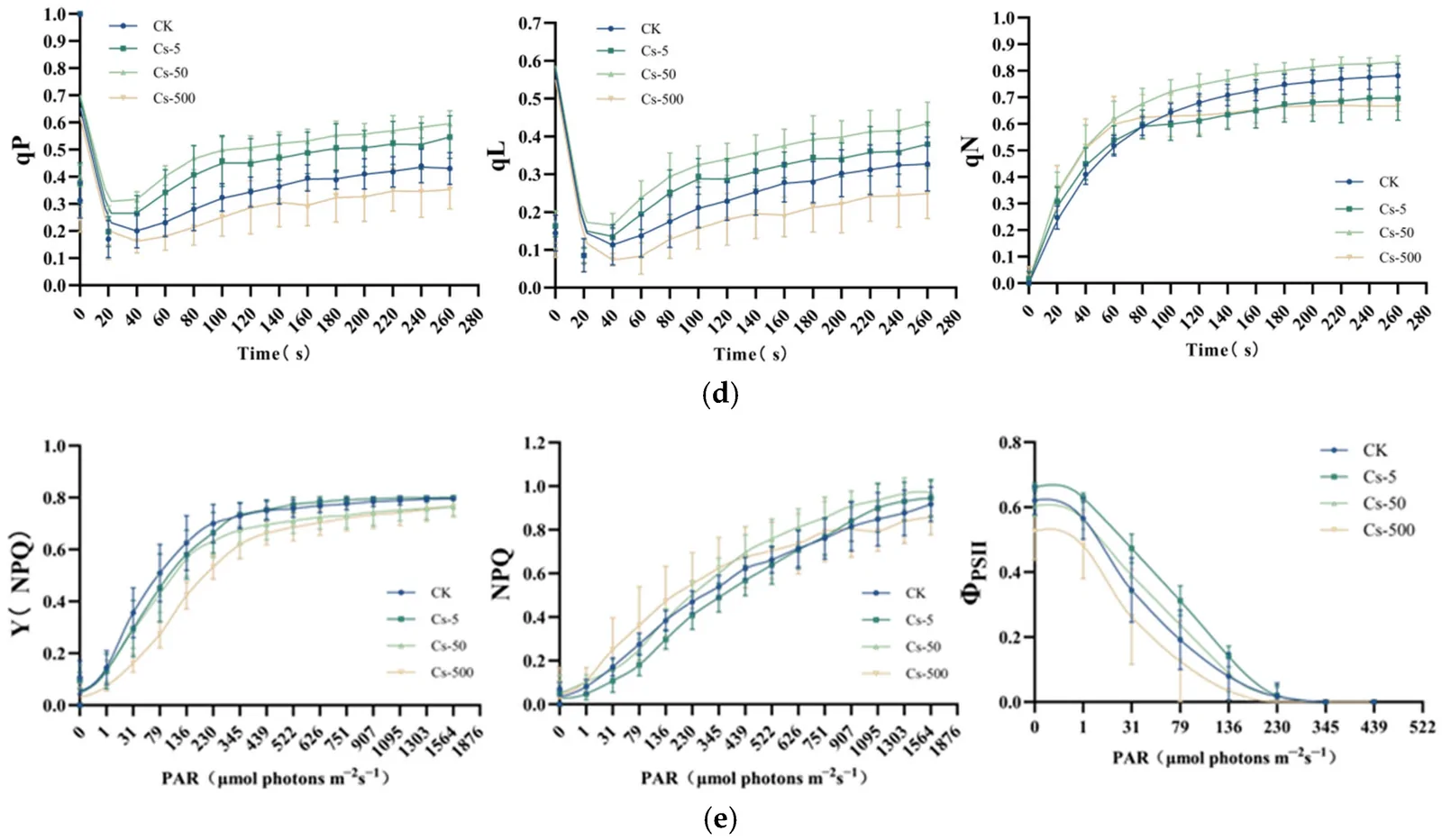
Photosynthetic physiological changes in *H. plumaeforme* under varying concentrations of cesium stress: (**a**) total chlorophyll content (Chl_total_); (**b**) box plot of *Fv*/*Fm*. Different lowercase letters indicate significant differences among treatments (one–way ANOVA with LSD post hoc multiple comparison, *p* < 0.05). (**c**) Chlorophyll fluorescence imaging, shown from top to bottom: *Fv*/*Fm*, *Fm*′, Y(NPQ), and *qN*. (**d**) Induction curves of *qP*, *qL*, and *qN*. (**e**) Light–response curves of Y(NPQ), NPQ, and *ΦPSII*. Data are mean ± SD (*n* = 6).

Cs^+^ stress significantly affected the photosynthetic physiology of *H. plumaeforme*, with all parameters exhibiting a concentration-dependent response. Total chlorophyll content (Chl_total_; Figure 3a) was highest in the Cs-5 group at 91.57 μg·mg^−1^FW, which was 115.3% significantly higher than that of CK (42.51 μg·mg^−1^FW). In the Cs-50 group, it was 52.50 μg·mg^−1^FW, still 23.5% above CK, showing an intermediate level. In the Cs-500 group, it dropped to 36.81 μg·mg^−1^FW, a decrease of 13.4% relative to CK. Chlorophyll a (Chl a) and chlorophyll b (Chl b) followed the same trend as Chl_total_, also increasing first and then decreasing.

Regarding chlorophyll fluorescence parameters, the maximum photochemical efficiency (*Fv*/*Fm*; Figure 3b) slightly increased by 1.6% in the Cs-5 group compared with CK, but was significantly decreased by 5.0% and 6.8% in the Cs-50 and Cs-500 groups, respectively. In the fluorescence imaging, the Cs-500 group showed distinct dark spots, while the maximum fluorescence yield (*Fm*′) imaging revealed large dark-red areas (Figure 3c). The light-response curves of the actual photochemical quantum efficiency (*ΦPSII*; Figure 3e) showed that *ΦPSII* in the Cs-5 group was higher than CK across all light intensities, with a 6.6% increase under dark-adapted conditions; the Cs-50 group exhibited a decreasing-then-increasing trend; and the Cs-500 group had the lowest *ΦPSII*, which was 15.4% lower than that of CK. In terms of photochemical quenching parameters (Figure 3d), the induction curves of the photochemical quenching coefficient (*qP*) and the photochemical quenching coefficient accounting for *ΦPSII* heterogeneity (*qL*) revealed that the Cs-50 group exhibited the largest increases, with *qP* and *qL* at 260 s being 38.3% and 32.6% higher than CK, respectively; in contrast, both *qP* and *qL* in the Cs-500 group decreased to the lowest values, with reductions of 18.1% and 24.0% relative to CK, respectively. Furthermore, in terms of non-photochemical quenching, the induction curve of the non-photochemical quenching coefficient (*qN*; Figure 3d) showed that *qN* was lowest in the Cs-500 group (decreased 14.6% vs. CK) and highest in the Cs-50 group (increased 6.7% vs. CK), while the Cs-5 group showed a moderate decrease (decreased 10.8% vs. CK); correspondingly, the fluorescence imaging of the Cs-500 group also displayed obvious and large-scale purplish-red areas (Figure 3c). The light-response curves of non-photochemical quenching (NPQ) showed that the Cs-500 group had the highest NPQ at low-to-medium light intensities, but it tended to be the lowest at the highest light intensity; in contrast, the quantum yield of regulated energy dissipation (Y(NPQ)) was the lowest in the Cs-500 group across all light intensities (Figure 3e), and its imaging appeared dark reddish-brown.

### 2.4. LC-MS Metabolomics Analysis of H. plumaeforme Under Cs^+^ Stress

**Figure 4:**
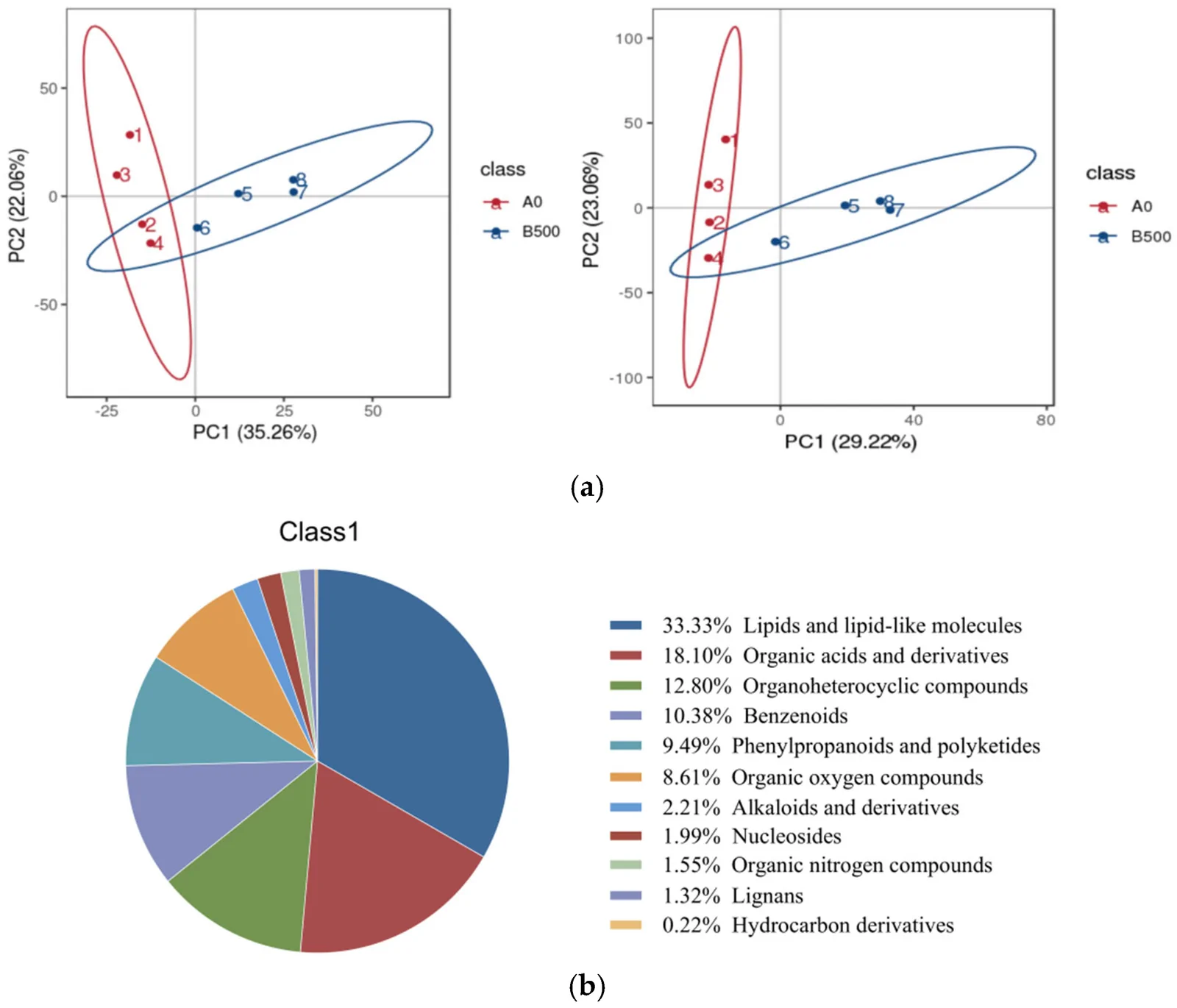
Cs−500 vs. CK groups in *H. plumaeforme*: (**a**) Principal component analysis (PCA). The left panel displays the PCA score plot in negative ion mode, and the right panel displays it in positive ion mode. In the score plots, the x–axis (PC1) and y–axis (PC2) correspond to the scores of the first and second principal components, respectively; different colored dots represent samples from different experimental groups, and the ellipse denotes the 95% confidence interval. The cumulative explained variance (between 50% and 60%) is considered acceptable in untargeted metabolomic studies, as biological variation in high-dimensional datasets is typically distributed across multiple factors rather than concentrated in the first few components [17]. (**b**) Pie chart of Class I classification of metabolites.

Untargeted metabolomics analysis was performed on *H. plumaeforme* in the Cs-500 and CK groups using liquid chromatography-mass spectrometry (LC-MS). A total of 1965 metabolites were identified in positive ion mode (pos) and 1172 metabolites in negative ion mode (neg; Table 2). Principal component analysis (PCA; Figure 4a; Folder S1 in Supplementary Materials) revealed that, within the 95% confidence interval, PC1 and PC2 explained 29.22% and 23.06% of the variance in positive ion mode, with a cumulative explanation rate of 52.28%; in negative ion mode, PC1 and PC2 accounted for 35.26% and 22.06% of the variance, with a cumulative rate of 57.32%. The score plots showed a clear separation trend between the Cs-500 and CK groups. Class 1 classification of the identified metabolites revealed that the most abundant category was Lipids and lipid-like molecules (33.33%), followed by Organic acids and derivatives (18.10%), Organoheterocyclic compounds (12.80%), and others (Figure 4b; Folder S2 in Supplementary Materials).

**Table 2:** Screening results of differential metabolites Cs-500 vs. CK groups in *H. plumaeforme.*

Compared Samples	Num. of Total Ident.	Num. of Total Sig.	Num. of Sig. Up	Num. of Sig. Down	Threshold
Cs-500 vs. CK pos	1965	81	41	40	*p*-value < 0.05, VIP > 1.0, FC > 1.5 or < 0.667
Cs-500 vs. CK neg	1172	79	26	53	

VIP refers to the Variable Importance in the Projection for the first principal component of the PLS-DA model [15] and the VIP value indicates the contribution of a metabolite to group discrimination. FC (Fold Change) is defined as the ratio of the mean quantitative values of each metabolite across all biological replicates between the compared groups. The *p*-value is calculated by the *t*-test [16] and represents the level of statistical significance of the difference.

**Figure 5:**
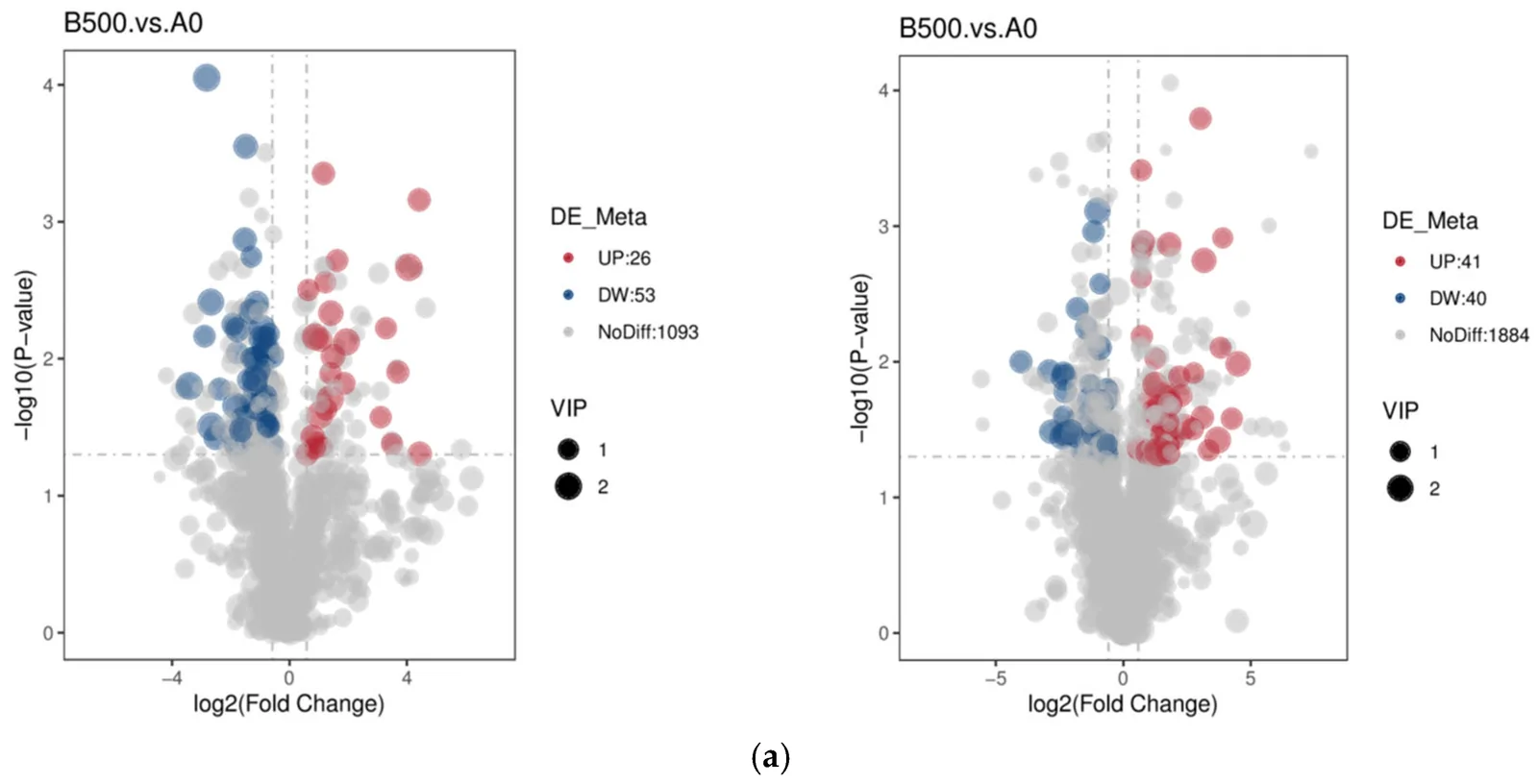

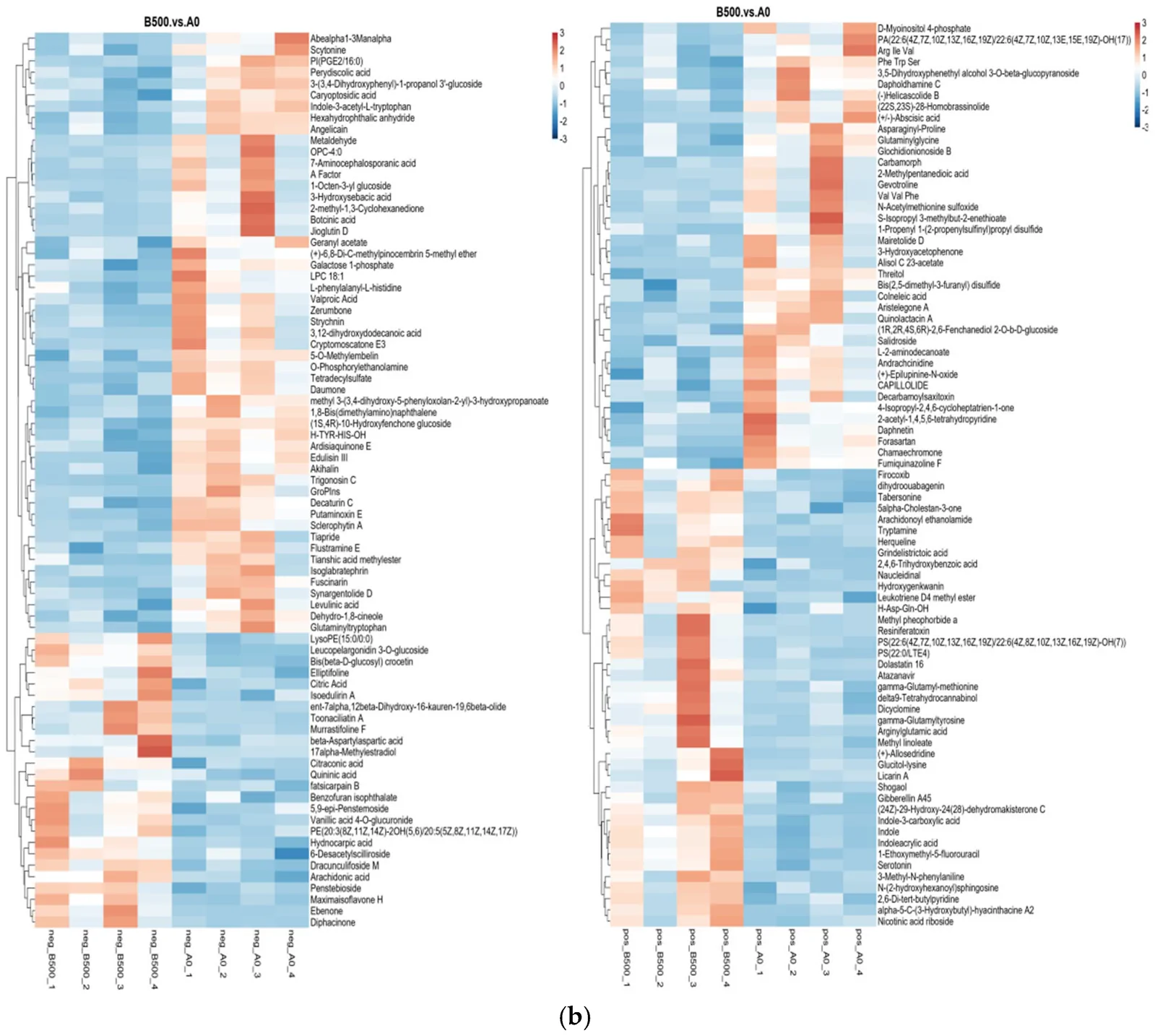
Differential metabolite profiles between the Cs−500 and CK groups in *H. plumaeforme*: (**a**) Volcano plot. The x–axis represents the fold change in metabolites between the two groups (log_2_(Fold Change)), and the y–axis represents the statistical significance level (−log_10_(*p*-value)). Each dot in the volcano plot corresponds to a metabolite; significantly upregulated metabolites are shown as red dots, significantly downregulated ones as blue dots, and dot size indicates the VIP value. (**b**) Clustering heatmap. The vertical axis represents sample clustering, and the horizontal axis represents metabolite clustering, with shorter dendrogram branches indicating higher similarity. The horizontal comparison reveals the clustering relationships of metabolite contents between groups. The left panel displays the negative ion mode, and the right panel displays the positive ion mode.

**Figure 6:**
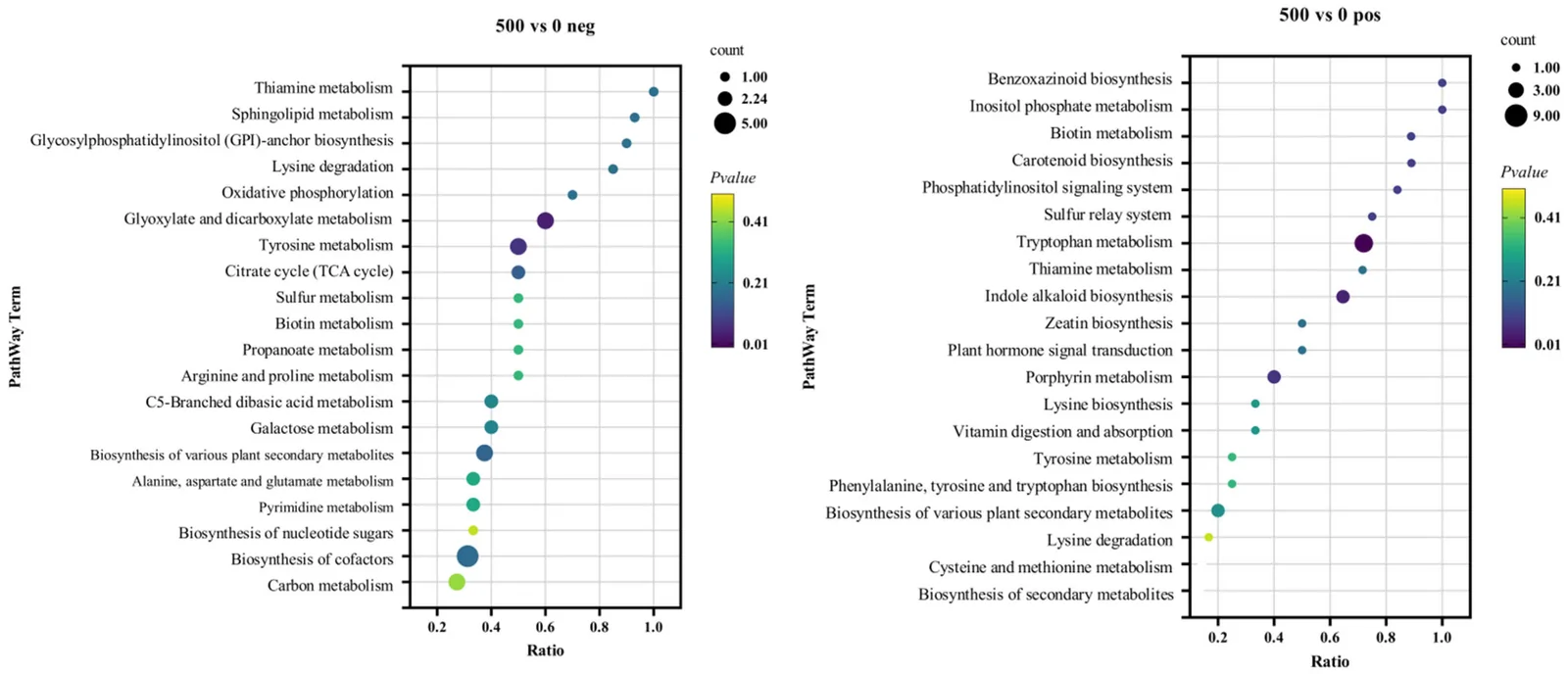
Bubble plot of KEGG-enriched pathways between the Cs-500 and CK groups in *H. plumaeforme*. The x-axis represents the ratio of the number of differentially abundant metabolites in a given pathway to the total number of identified metabolites in that pathway (i.e., x/y); a higher ratio indicates a greater degree of enrichment. The color of each bubble indicates the *p*-value from the hypergeometric test, with smaller *p*-values denoting higher reliability and greater statistical significance. The size of each bubble reflects the number of differentially abundant metabolites in the corresponding pathway, with larger bubbles representing more metabolites. The left panel displays the negative ion mode, and the right panel displays the positive ion mode.

Using the criteria of variable importance in projection (VIP) > 1.0, fold change (FC) > 1.5 or FC < 0.667, and *p* < 0.05 as screening thresholds, a total of 160 significantly differential metabolites were identified (Table 2). Among these, 79 metabolites were detected in negative ion mode, with 53 downregulated and 26 upregulated (downregulated being 2.04 times the upregulated); in positive ion mode, 81 metabolites were identified, with 41 upregulated and 40 downregulated, showing a roughly balanced ratio. Significantly upregulated metabolites included defensive secondary metabolites such as sesquiterpene lactones (Ebenone, 17α-Methylestradiol) and flavonoids (Maximaisoflavone H). In contrast, significantly downregulated metabolites included acidic or carboxyl-containing compounds such as phospholipid precursors (O-Phosphorylethanolamine), diterpenoids (Trigonosin C), and hydroxy acid derivatives (Boticinic acid). Meanwhile, basic or nitrogen-containing metabolites exhibited a relatively balanced ratio of up- to downregulation (Figure 5a,b; Folders S3 and S4 in Supplementary Materials). KEGG enrichment analysis (hypergeometric test, *p* < 0.05) (Figure 6; Folder S5 in Supplementary Materials) revealed that pathways including Tryptophan metabolism (*p* = 0.0009), Indole alkaloid biosynthesis (*p* = 0.0464), and Glyoxylate and dicarboxylate metabolism (*p* = 0.0424) were significantly enriched. Within the tricarboxylic acid (TCA) cycle and oxidative phosphorylation pathways, Phosphoenolpyruvate (PEP) and Succinate were significantly downregulated, while Citrate was significantly upregulated. Central carbon metabolic intermediates such as Citric acid and Quinic acid accumulated, whereas (S)-Malic acid showed no significant change. In the tryptophan metabolic pathway, Indole-3-ethanol, Tryptamine, Indole, and Serotonin were all significantly upregulated, whereas Melatonin and its downstream product 6-Hydroxymelatonin showed no significant differences.

## 3. Discussion

### 3.1. Coordinated Collapse of ROS Metabolic Imbalance and the Osmotic Regulatory Network

In the present This study revealed that the morphological damage in *H*. *plumaeforme* under cesium (Cs^+^) stress was closely associated with a progressive collapse of the intracellular redox balance. Under low-concentration (5 mg·L^−1^) Cs^+^ treatment, H_2_O_2_ content increased first and reached its peak, accompanied by a significant accumulation of the lipid peroxidation product MDA, indicating that the membrane system had already been attacked oxidatively even without obvious morphological damage. At this stage, O_2_·^−^ was not yet generated in large quantities, while CAT activity exhibited an inverse trend relative to H_2_O_2_ content, suggesting that the scavenging capacity of CAT was relatively insufficient [18], thereby allowing H_2_O_2_ to accumulate and potentially serve as a signal for downstream defense responses [19,20,21]. When the Cs^+^ concentration was increased to 50 mg·L^−1^, elevated the production rate and content of O_2_·^−^ reached their maxima, with a corresponding surge in SOD activity, reflecting the rapid dismutation of O_2_·^−^ to H_2_O_2_. However, POD activity, after peaking at the low concentration, decreased continuously, and GSH content was already markedly depleted at 5 mg·L^−1^ and remained at a low level upon entering the 50 mg·L^−1^ stage. This directly constrained the GSH-dependent GPX-mediated scavenging of lipid peroxides, causing MDA content to decrease but still remain above the control level [22,23]. At this point, ASA and DHA contents increased substantially, partly offsetting oxidative stress via the ASA-GSH cycle [24]; however, GPX activity was continuously suppressed with increasing Cs^+^ concentration, indicating a functional impairment of the enzymatic lipid peroxide scavenging system. At the highest concentration (500 mg·L^−1^), the ·OH content increased sharply, and its strong oxidizing power directly damaged membrane lipids, proteins, and nucleic acids, ultimately leading to irreversible damage, including loosely arranged stem cells, ruptured cell walls, and cavity formation [25]. This sequential shift in the dominant ROS species from H_2_O_2_ to O_2_·^−^ and then to ·OH is consistent with the oxidative cascade patterns previously reported in mosses under non-essential metal stress [26,27]. The present study delineated the concentration-dependent threshold characteristics of different ROS components along the Cs^+^ concentration gradient, unveiling the phased and irreversible progression of oxidative damage in mosses under heavy metal stress [28].

The exacerbation of oxidative damage occurred concurrently with the dysregulation of the osmotic regulatory network. Free proline content showed a continuous accumulation trend with increasing Cs^+^ concentration, reaching a level 2.8-fold higher than that of the control at 500 mg·L^−1^ [29]. The accumulation of proline not only serves as an osmotic regulator and a scavenger of ·OH, but also reflects the cellular redox imbalance and the disturbance of carbon and nitrogen metabolism [30]. Soluble sugar content exhibited an increasing-then-decreasing pattern, peaking at 50 mg·L^−1^ and then declining at 500 mg·L^−1^. This indicates that under moderate stress, photosynthetic products were still sufficient to meet osmotic adjustment demands, whereas under high-level stress, carbon assimilation was impaired or respiratory consumption was enhanced, resulting in a reduction in carbohydrate reserves [29,31]. Soluble protein content decreased initially and then increased, which may be attributable to oxidative cross-linking or degradation at the early stage, and to the induced synthesis of stress proteins such as heat shock proteins at the later stage [32]. In summary, under low Cs^+^ concentration, *H. plumaeforme* maintained cellular homeostasis through upregulation of the ASA-GSH cycle and initial accumulation of osmotic regulatory substances. As the concentration increased, however, GPX inhibition, GSH depletion, and ·OH burst led to the breakdown of the antioxidant system, while osmotic regulation also became disordered due to the restricted supply of carbon skeletons, ultimately resulting in cellular structural disintegration [19,30]. Taken together, these results delineate a “hierarchical regulatory failure cascade”: the early H_2_O_2_ burst triggers antioxidant upregulation and osmotic solute accumulation as a compensatory attempt; however, progressive GSH depletion and GPX inhibition, coupled with the transition to ·OH-dominated oxidative species, overwhelm the cellular defense capacity, leading to the coordinated collapse of both redox homeostasis and osmotic adjustment [18]. This integrative view underscores that the tolerance threshold of *H. plumaeforme* to cesium lies between 50 and 500 mg·L^−1^, and that the coordinated collapse of the osmotic-antioxidant network constitutes the intrinsic biochemical basis of the observed morphological damage.

### 3.2. Photosynthetic Hormesis at Low Cs^+^ and Photoprotection Collapse at High Cs^+^

Photosynthetic parameters of *H. plumaeforme* exhibited a typical biphasic dose response to Cs^+^ stress, with stimulation at low concentrations and inhibition at high concentrations [33]. Treatment with 5 mg·L^−1^ Cs^+^ increased total chlorophyll content by 115.3% relative to the control, slightly elevated *Fv*/*Fm*, and resulted in higher *ΦPSII* and photochemical quenching coefficients *qP* and *qL* than those of the control [34]. This low-dose stimulation phenomenon can be explained by hormesis, whereby low-level stress factors over-activate repair and anabolic pathways [35,36,37]. As a chemical analog of potassium, cesium might transiently stimulate chloroplast development or thylakoid membrane stacking by partly replacing K^+^, while low-concentration H_2_O_2_ may also act as a signaling molecule involved in upregulating the expression of genes related to chlorophyll synthesis [30,38,39]. When Cs^+^ concentration was increased to 50 mg·L^−1^, *Fv*/*Fm* began to decline, and the light-response curve of *ΦPSII* showed lower values than the control under low light intensity, indicating mild photoinhibition of *PSII* [33]. However, *qP* and *qL* could still increase under certain light intensities, suggesting that photochemical electron transport could be partially compensated by enhanced QA oxidation [40].

Notably, upon exposure to 500 mg·L^−1^ Cs^+^, chlorophyll content dropped below the control level, *Fv*/*Fm* decreased significantly, *ΦPSII* was the lowest across all light intensities, and *qP* and *qL* were greatly reduced, indicating that the open proportion of *PSII* reaction centers was severely insufficient and the electron transport chain was blocked [33]. Meanwhile, *qN*, which is related to non-photochemical energy dissipation, decreased, while Y(NPQ) remained at the lowest level across all light intensities, and the light-response curve of NPQ tended to stagnate under high light. Fluorescence imaging revealed large dark spots and purplish-red areas, indicating that the establishment of the trans-thylakoid proton gradient was impaired and the *ΔpH*-dependent thermal dissipation (*qE*) mechanism was inactivated [41,42]. Furthermore, ·OH content increased sharply at this concentration. Since ·OH can specifically attack the D1 protein in the *PSII* reaction center, inhibit the degradation and de novo synthesis of damaged D1, and consequently block the *PSII* repair cycle [43], the collapse of the photosynthetic system under high-concentration Cs^+^ is not only a consequence of oxidative stress, but also exacerbates excitation pressure through the inhibition of electron transport, forming a positive feedback loop of photoinhibition, ROS generation, and repair blockage [44]. This photoinhibition-ROS feedback loop is intimately linked to the metabolic impairments observed in central carbon metabolism, as the ATP and NADPH deficits resulting from electron transport blockade further compromise the carbon fixation and energy supply required for repair processes [40]. Similar photoprotection collapse has been reported in vascular plants under heavy metal stress [45,46], and our findings demonstrate that such an energy disaster cascade effect also exists in mosses, despite their structurally simple organization.

### 3.3. Central Carbon Impairment and Defensive Tryptophan Shift Under High Cs^+^

Untargeted LC-MS-based metabolomics analysis provided a metabolic-level explanation for the physiological damage induced by high-concentration Cs^+^ stress. Significant differential metabolites included downregulation of Phosphoenolpyruvate (PEP) and Succinate and upregulation of Citrate and Quinic acid, pointing to a TCA cycle bottleneck at succinate dehydrogenase or α-ketoglutarate dehydrogenase [47]. The blockage of key nodes in the TCA cycle would reduce the production of NADH and ATP, which is consistent with the observed decrease in soluble sugar content and the deterioration of cellular energy status under high-concentration Cs^+^ [48,49]. The significant enrichment of the Glyoxylate and dicarboxylate metabolism pathway suggested that cells initiated anaplerotic reactions to replenish TCA cycle intermediates [50,51]; however, this also implied that carbon flux was diverted away from biosynthesis, further restricting the supply of carbon skeletons required for growth. On the other hand, the Tryptophan metabolism pathway showed the most prominent enrichment, with Indole-3-ethanol, Tryptamine, Indole, and Serotonin all being significantly upregulated [52]. Tryptophan and its indole derivatives serve as common precursors for auxin (Indole-3-acetic acid) and various defensive secondary metabolites [53]. The accumulation of Serotonin may be involved in cell wall lignification or suberization to reinforce the physical barrier, whereas Melatonin and its downstream metabolite 6-Hydroxymelatonin showed no significant changes, indicating that the Tryptophan metabolic flux was preferentially directed toward the Serotonin branch rather than the Melatonin pathway [54,55].

Meanwhile, defensive secondary metabolites such as flavonoids (e.g., Maximaisoflavone H) and sesquiterpene lactones were significantly upregulated, and these compounds have been demonstrated to possess direct ROS-scavenging and metal-chelating functions [56,57]. The downregulation of phospholipid precursors (O-Phosphorylethanolamine) and acidic metabolites suggested that membrane phospholipid composition underwent remodeling and that intracellular pH homeostasis might have been disrupted, which is consistent with the observed damage to the membrane system and the loss of cellular compartmentalization [58]. Notably, the above metabolic changes were not merely passive reflections of toxicity, but rather represented a strategy of preferentially allocating limited carbon and nitrogen resources to chemical defense and membrane protection under energy-limited conditions [59]. However, this defense at the expense of growth ultimately failed to prevent the disintegration of cellular architecture. To explicitly bridge the physiological and metabolomic datasets, we further examined the correlative relationships between key metabolites and physiological indices. The accumulation of Serotonin and flavonoids occurred concurrently with the elevated SOD and POD activities, suggesting a synergistic engagement of metabolic defense and enzymatic antioxidant systems [52]. Conversely, the downregulation of PEP and Succinate coincided with the decline in chlorophyll content and *Fv*/*Fm*, implying that TCA cycle impairment may directly contribute to photosynthetic energy limitation [60]. Therefore, we propose that *H. plumaeforme* under cesium stress does not rely solely on its intrinsic antioxidant and osmotic adjustment mechanisms, but rather attempts to maintain a transient homeostasis through widespread reprogramming of primary and secondary metabolic pathways. This provides new insights into the tolerance strategies of mosses in response to heavy metal stress.

### 3.4. Feasibility and Limitations of H. plumaeforme as a Cesium Bioindicator

Synthesizing multi-level evidence from morphology, physiological biochemistry, and metabolomics, *H. plumaeforme* shows potential as a bioindicator plant for cesium contamination. Previous studies using moss bags to monitor radioactive cesium in the environment after the Fukushima nuclear accident have confirmed that mosses possess high efficiency in cesium accumulation and long-term retention, and the strong cation-exchange capacity of *H. plumaeforme* is consistent with these reported accumulation patterns [11,61]. Its responses exhibit a clear concentration-dependence: at 5 mg·L^−1^ Cs^+^, significant increases in H_2_O_2_ and MDA were already triggered, which occurred prior to visible morphological changes, thus serving as sensitive biochemical endpoints for early warning; at 50 mg·L^−1^, O_2_·^−^ burst and mild suppression of photochemical parameters could indicate moderate pollution stress; and at 500 mg·L^−1^, severe tissue damage and metabolic network collapse marked the tolerance limit. Comparison with other mosses reveals characteristic features of Cs responses. In *H. cupressiforme*, a species widely employed in heavy metal biomonitoring, exposure to non-essential metals typically induces a rapid and sustained increase in SOD and CAT activities [62], whereas *H. plumaeforme* shows a more transient and concentration-limited antioxidant response, with GPX activity continuously suppressed at higher Cs^+^ levels, suggesting a lower enzymatic buffering capacity against severe oxidative challenge. In contrast, *Racomitrium japonicum*, a moss known for its exceptional tolerance to ionizing radiation and heavy metals, maintains high constitutive levels of antioxidants and displays an efficient *PSII* repair cycle under stress [31,33]; *H. plumaeforme* appears less resilient in this regard, as evidenced by the irreversible photoprotection collapse at 500 mg·L^−1^ Cs^+^. Compared with *Tortella tortuosa*, a desiccation-tolerant species that employs strong cell wall-based cation sequestration and metabolic quenching mechanisms [51], *H. plumaeforme* shares a similar reliance on tryptophan-derived defensive metabolites but lacks the robust cell wall thickening that may enhance metal immobilization. These interspecific differences highlight that the response strategies of *H. plumaeforme* are neither universal nor inferior; this moderate tolerance, combined with sensitive early biochemical responses, makes it particularly suitable for early-warning bioindication. Meanwhile, indicators such as proline, H_2_O_2_, and chlorophyll fluorescence parameters can be rapidly determined with routine laboratory equipment, making them suitable for large-scale screening [26].

It should be noted that the hydroponic concentrations used in this study are higher than those in most natural water bodies, but may correspond to cesium levels in locally highly contaminated habitats, such as pooled water around leakage points, or in soil pore water. For practical application, a calibratable evaluation model should be developed by establishing quantitative relationships between tissue cesium residues and physiological response indicators. Furthermore, the differential responses of Tryptophan metabolism and TCA cycle intermediates revealed by metabolomic analysis provide potential biomarkers for constructing a cesium-specific metabolic profile and for distinguishing cesium stress from other metal stresses, which aligns with the growing trend of applying environmental metabolomics in pollution diagnosis. Overall, *H. plumaeforme* can be integrated into a tiered monitoring framework, in which rapid biochemical assays are first used for early warning, followed by chemical analysis to confirm cesium load, thereby enabling efficient and cost-effective spatial assessment of cesium contamination.

It should be acknowledged that this study has several limitations, although these constraints do not compromise the reliability of the core conclusions. First, the experiment was conducted under controlled hydroponic conditions with a single Cs^+^ treatment solution, which did not reflect the inhibitory effects of competing cations such as K^+^ and NH_4_^+^ on cesium uptake in a natural environment [63] nor did it simulate realistic scenarios such as wet–dry cycles or long-term low-dose exposure. Therefore, caution is required when directly extrapolating the concentration-effect thresholds obtained in this study to complex field habitats. The exposure regime under hydroponic conditions differs fundamentally from the transport and transformation processes in soil–plant systems, and the former may overestimate the bioavailability and physiological effects of cesium. Second, this study focused on a single population of *H. plumaeforme* and lacked taxonomic comparisons with other mosses under Cs^+^ stress [64], which to some extent limits the generalizability of our conclusions to the broader bryophyte group. Given that different moss taxa may exhibit significant interspecific variations in cell wall composition, cation exchange capacity, and antioxidant capacity, future studies should expand species coverage to validate the representativeness of the response patterns revealed herein. Third, the metabolomic analysis only compared the control group with the highest-concentration treatment group and lacked intermediate concentration gradients and time-series data, making it difficult to capture the dynamic evolution of the metabolic network from adaptive adjustment to irreversible damage. Fourth, the present study did not determine the actual cesium accumulation or the bioconcentration factor in *H. plumaeforme*, which constrains a deeper understanding of the quantitative relationship between tissue burden and physiological responses. Given these limitations, addressing them will be a key priority in our future research.

## 4. Materials and Methods

### 4.1. Experimental Materials and Design

The collected *H. plumaeforme* plants were rinsed with deionized water to remove surface debris, then spread as intact mats in plastic trays and placed in a glass greenhouse for acclimation. According to the natural habitat conditions of the species, the plants were pre-acclimated for 7 days. Thereafter, healthy gametophytes of uniform growth (10 g fresh weight per group) were selected, transferred to plastic culture boxes, and subjected to stress treatments at 25 °C and a relative light intensity of 60%. From 13:30 to 16:30 each day, 70 mL of freshly prepared CsCl solution was added to each culture box to thoroughly immerse the gametophyte stems and leaves. After each treatment, the plants were promptly separated from the solution to terminate the stress. The stress treatment lasted for 7 consecutive days, a duration widely adopted in moss stress studies and validated by our preliminary experiments, being sufficient to induce time-dependent antioxidative and photosynthetic responses while avoiding confounding effects such as nutrient depletion and population growth that may arise from prolonged culture [13,29,65]. The experiment included a control group (CK, receiving an equal volume of deionized water instead of CsCl solution) and three treatment groups with CsCl concentrations of 5, 50, and 500 mg·L^−1^, designated as Cs-5, Cs-50, and Cs-500 in the figures and tables. Each treatment was performed with three biological replicates.

### 4.2. Observation of Phenotypic and Cellular Characteristics

For growth status observation, the *H. plumaeforme* plants were rinsed with distilled water, then arranged in order of concentration (CK, 5, 50, and 500 mg·L^−1^) on a white specialized background cloth. Macro-photographs were taken using a *Canon* camera. For cellular morphology observation (paraffin sections), cleaned samples were fixed in FAA for ≥24 h, dehydrated in graded ethanol, cleared in xylene, and infiltrated with a xylene-paraffin gradient. After embedding in cassettes, 8-μm sections were cut, floated on adhesive slides at 38 °C for natural flattening, dewaxed in xylene, ethanol series, and water, then stained with safranin-fast green and examined microscopically. For scanning electron microscopy (SEM) observation, an appropriate amount of *H. plumaeforme* was weighed, cleaned, and dried at 70 °C to constant weight. After grinding with liquid nitrogen, the samples were sent to the Analytical and Testing Center of Southwest University of Science and Technology for examination of the surface morphology.

### 4.3. Measurement of Antioxidant Physiological Parameters

H_2_O_2_ content (potassium iodide method [66]) was determined by mixing the supernatant with 10 mM PBS7.0 and 1 M KI, followed by incubation in the dark at room temperature for 1 h and absorbance measurement at 390 nm. Histochemical localization of H_2_O_2_ (DAB staining method [67]) was performed by incubating tissues with 1 mg·mL^−1^ DAB solution in the dark for 8 h, then destained in ethanol and examined microscopically. O_2_·^−^ content and production rate (hydroxylamine hydrochloride method [68]) were assayed by adding 0.05 M PBS7.8 and 1 mM hydroxylamine hydrochloride to the supernatant, incubating at 25 °C for 1 h, followed by color development with 17 mM *p*-aminobenzenesulfonic acid and 7 mM α-naphthylamine at 25 °C for 20 min, and absorbance was read at 530 nm. O_2_·^−^ distribution (NBT reduction method [69]) was visualized by incubating tissues with 1 mg·mL^−1^ NBT solution for 12 h, then destained and observed microscopically. ·OH content (deoxyribose degradation method [70]) was measured by grinding samples with 0.015 M 2-deoxy-D-ribose, reacting the supernatant with 0.5% (w/v) TBA in acetic acid at 37 °C for 2 h, followed by a boiling water bath for 30 min, and absorbance was recorded at 532 nm. Activities of SOD, POD, and CAT were determined using kits purchased from Nanjing Jiancheng Bioengineering Institute. MDA content (TBA colorimetric method [71]) was assayed by mixing the supernatant with 0.67% TBA solution, heating in a boiling water bath for 30 min, cooling, centrifuging, and measuring absorbance at 450, 532, and 600 nm.

ASA and DHA contents (spectrophotometric method [72]) were determined as follows: the supernatant was mixed with 5% TCA and acetic acid, then sequentially with 0.4% phosphoric acid, 0.5% 4,7-diphenyl-1,10-phenanthroline (BP), and 0.03% FeCl_3_. After incubation at 30 °C for 90 min, absorbance was read at 534 nm for ASA content. For total ASA (T-ASA) determination (spectrophotometric method with DTT reduction), the supernatant was treated with 60 mM DTT in ethanol, adjusted to pH 7–8 with Na_2_HPO_4_-NaOH, incubated at room temperature for 10 min, then adjusted to pH 1–2 with 20% TCA, and measured following the same procedure. DHA content was calculated as the difference between T-ASA and ASA. GSH content (DTNB method [73]) was assayed by mixing the supernatant with 0.1 M PBS7.7 and 4 mM DTNB. After 10 min at room temperature, absorbance was immediately read at 412 nm; an equal volume of PBS6.8 was used as the blank. GPX activity (DTNB method [74]) was measured by reacting the supernatant with 1 mM GSH and 1.5 mM pre-heated H_2_O_2_ at 37 °C for 3 min. The reaction was stopped by adding 1.67% metaphosphoric acid, and after centrifugation, the supernatant was mixed with 0.32 mM Na_2_HPO_4_ and DTNB. After 5 min, absorbance was read at 412 nm.

Soluble protein content (Coomassie Brilliant Blue method [75]) was determined by mixing the supernatant with G-250 solution, allowing it to stand for 2 min, and measuring absorbance at 595 nm. Free proline content (ninhydrin method [76]) was assayed by cutting samples into small pieces and extracting with 3% sulfosalicylic acid in a boiling water bath for 10 min. The supernatant was then mixed with glacial acetic acid and acidic ninhydrin, heated in a boiling water bath for 40 min until a red color developed, followed by extraction with toluene and absorbance measurement at 520 nm. Soluble sugar content (anthrone method [77]) was measured by heating the extract in a boiling water bath for 30 min, cooling, filtering, and adjusting to volume. An aliquot of the extract was mixed with distilled water and anthrone reagent, then concentrated sulfuric acid was slowly added. After heating in a boiling water bath for 10 min and cooling, absorbance was read at 620 nm.

### 4.4. Measurement of Photosynthetic Parameters

Chlorophyll content was determined by the ethanol-acetone method [78]. Fresh samples (0.5 g) were cut into small pieces, placed in a light-proof container, and mixed with 10 mL of 50% ethanol-acetone (1:1) solution. The tube was sealed and vigorously shaken for 1–2 min, then extracted in the dark at room temperature for 24 h. After centrifugation at 4000 rpm for 10 min, 200 μL of the supernatant was used to measure absorbance at 645 and 663 nm. Chlorophyll content was calculated as follows:

Chl a = (12.72 × OD663 − 2.59 × OD645) × V/W(1)

Chl b = (22.88 × OD645 − 4.67 × OD663) × V/W(2)

Chl_total_ = Chl a + Chl b (3)

where V is the total volume of extract (mL); W, fresh weight of the sample (g).

Chlorophyll fluorescence parameters were measured using the ImagingWin system. Within 24 h after treatment, multiple *H. plumaeforme* shoots were randomly selected. The measuring light intensity was adjusted to the actinic light intensity, and the samples were dark-adapted for 15–20 min. *PSII* parameters, including *ΦPSII*, *Fv*/*Fm*, NPQ, Y(NPQ), and Y(NO), were determined using a chlorophyll fluorometer equipped with a probe. For chlorophyll fluorescence imaging, an Image-PAM fluorometer was used. Uniform and healthy plants were selected, and images were captured after adjusting parameters according to the instrument conditions.

### 4.5. Metabolomic Analysis

Samples from the CK and Cs-500 groups were collected, thoroughly rinsed with distilled water, bagged and labeled, then immersed in liquid nitrogen for at least 15 min. They were subsequently transferred to a cryobox containing dry ice and sent to Novogene Co., Ltd. (Beijing, China). for untargeted LC-MS metabolomics analysis.

### 4.6. Data Processing and Statistical Analysis

The experimental data were processed and statistically analyzed using Microsoft Excel and SPSS 27 software. All data are presented as mean ± standard deviation (SD) from independent experiments. Graphs, including bar charts, box plots, and curve plots, were generated using GraphPad Prism Version 10.4.1.

## 5. Conclusions

This study systematically elucidated the physiological and metabolic response mechanisms of the moss *H. plumaeforme* under cesium (Cs^+^) stress through integrated analyses of morphology, antioxidant physiology, photosynthetic performance, and untargeted metabolomics. The results demonstrated that the responses of *H. plumaeforme* to Cs^+^ were strongly concentration-dependent. Low Cs^+^ exposure induced an early oxidative signaling response characterized by H_2_O_2_ accumulation and mild stimulation of photosynthetic activity, whereas moderate stress enhanced superoxide production and activated antioxidant defense mechanisms. In contrast, high Cs^+^ exposure caused severe cellular deformation, excessive hydroxyl radical accumulation, inhibition of photosystem II, disruption of the coordinated antioxidant–osmotic regulatory network, and ultimately irreversible cellular damage. Metabolomic analysis further revealed that severe Cs^+^ stress substantially reprogrammed cellular metabolism. The inhibition of central carbon metabolism, together with the enrichment of tryptophan metabolism and accumulation of defensive secondary metabolites, indicated a metabolic shift from growth-related processes toward stress defense. Integrating physiological and metabolic evidence, we propose that cesium toxicity in *H. plumaeforme* follows a progressive cascade involving oxidative imbalance, photosynthetic dysfunction, metabolic reprogramming, and structural collapse. Importantly, several physiological indicators, including H_2_O_2_ accumulation, chlorophyll fluorescence parameters, and osmotic regulatory substances, responded sensitively before obvious morphological damage occurred, highlighting their potential as early-warning biomarkers of cesium stress.

Overall, this study demonstrates that the response of *H. plumaeforme* to cesium is not governed by a single antioxidant pathway, but by the coordinated regulation of redox homeostasis, photosynthetic function, osmotic adjustment, and metabolic reprogramming. This integrated response underpins both its tolerance strategy and its potential application as a sensitive bioindicator for environmental cesium contamination.

## Figures and Tables

**Table 1:** Contents of soluble protein, soluble sugar, and free proline of *H. plumaeforme* under varying concentrations of cesium stress.

Cs^+^ (mg·L^−1^)	Soluble Protein Content (mg·g^−1^FW)	Soluble Sugar Content (mg·g^−1^FW)	Free Proline Content (μg·g^−1^FW)
0	6.288 ± 0.119 c	64.432 ± 1.378 d	6.651 ± 0.821 d
5	5.873 ± 0.179 a	72.606 ± 0.218 c	7.409 ± 0.221 c
50	6.517 ± 0.148 b	103.652 ± 1.405 a	14.563 ± 0.150 b
500	6.865 ± 0.163 a	76.129 ± 0.432 b	18.848 ± 0.719 a

Data are mean ± SD (*n* = 6). Different lowercase letters indicate significant differences among treatments (one-way ANOVA with LSD post hoc multiple comparison, *p* < 0.05).

## Data Availability

Data will be made available on request.

## Supplementary Materials

The following supporting information can be downloaded at: https://www.mdpi.com/article/10.3390/plants15162504/s1; the complete metabolomics dataset. Folder S1: dadasheet for Figure 4a; Folder S2: dadasheet for Figure 4b; Folder S3: dadasheet for Figure 5a; Folder S4: dadasheet for Figure 5b; Folder S5: dadasheet for Figure 6.
